# Admission Pattern and Treatment Outcome in Pediatric Intensive Care Unit, Tertiary Hospital, Addis Ababa, Ethiopia

**DOI:** 10.4314/ejhs.v32i3.4

**Published:** 2022-05

**Authors:** Gemechu Edae, Atnafu Mekonnen Tekleab, Melaku Getachew, Tigist Bacha

**Affiliations:** 1 Assistant Professor of Pediatrics and Child Health, Department of Pediatrics and Child Health, Arsi University, School of Medicine, Oromia, Ethiopia; 2 Associate Professor of Pediatrics and Child Health, Department of Pediatrics and Child Health, Saint Paul's Hospital Millennium Medical College, Addis Ababa, Ethiopia; 3 Assistant Professor of Emergency and Critical Care Medicine, Department of Emergency and Critical Care Medicine, Haramaya University, School of Medicine, Harar, Ethiopia; 4 Associate Professor, Pediatric Emergency and Critical Care Medicine, Pediatric Cardiac intensivist, Department of Pediatrics and Child Health, Saint Paul Millennium Medical College, Addis Ababa, Ethiopia

**Keywords:** Low resource countries, Pattern, Intensive care unit, outcome

## Abstract

**Background:**

Knowledge of the clinical profile and outcomes of critically ill children admitted to Pediatric Intensive Care Unit (PICU) in developing countries aids with the identification of priorities and the resources needed to improve the outcome of critically ill patients. This study aimed to assess the admission pattern, outcomes, and associated factors of patients admitted to the PICU of St Paul's Hospital Millennium Medical College, Addis Ababa, Ethiopia.

**Methods:**

Institutional-based cross-sectional study was done. Data was collected through chart abstraction from patients admitted to the PICU between January 2017 and December 2018. SPSS 20.0 was used to analyze the data. Descriptive statistics, cross-tabulations, and logistic regressions were used.

**Results:**

A total of 260 pediatric patients were analyzed. The mean age at admission was 48.13 ± 53.65 months, with M: F ratio of 1.4:1. The mean and median duration of PICU stay was 7.26 ±6.87 days, and 6.0 days respectively. The most commonly affected organ systems were the central nervous system (79, 33.2%) and respiratory system (55, 23.1%). Mechanical ventilation and admission after cardiopulmonary resuscitation (p < 0.001) were independent predictors of mortality. Infectious causes of illnesses were the leading causes of admission and death in the PICU.

**Conclusion:**

The mortality rate of our PICU was 21.1 %. In this study, post-cardiopulmonary resuscitation admission and use of mechanical ventilation were statistically significant predictors of mortality indicating the need for well equipping and staffing the PICU to improve the outcome of such critically sick patients.

## Introduction

A pediatric intensive-care unit (PICU) is one of the hospital units, where critically ill pediatric patients are admitted. It requires advanced airway, hemodynamic and organ supports for critically ill children to achieve an outcome better than if the patients were admitted into other parts of the hospital. Over the last three decades, the profession of intensive care has evolved tremendously. Advances in the field of intensive care medicine have greatly improved the care of critically ill patients (1, 2).

The intensive care community in resource-rich countries has amassed great expertise and invested significant resources in the care of critically sick patients (3). Intensive care medicine is a developing discipline in resource-limited countries (RLC). Financial limitations due to inadequate insurance and national health systems together with severe logistic and educational problems account for high morbidity and mortality rates in ICUs of RLC (4).

Patients' reported disease characteristics and outcomes differ greatly from one population to the others. Also, the ICU mortality varies from setting to setting (5–11). There is an increased risk of mortality for a child admitted to PICU of RLC country than a child admitted to PICU of a resource-rich country due to differences in structure and staffing of the PICUs of the two settings (12). The variation in PICU mortality rate is also observed among hospital settings of developing countries for example 27.1% mortality rate reported from South Africa (13) and 12.9% mortality rate reported from Pakistan (14). Similarly, within the same country, the PICU mortality rate varies from center to center. In Ethiopia, the PICU mortality rate was 8.5% from Ayider Hospital (9), 40.0% from Jimma University hospital (10), and 32.6% from Gondar Hospital (11).

Factors such as the reason of admission, need for mechanical ventilation, Glasgow coma scale, need for inotrope, cardiac arrest during admission, non-reactive pupil on admission, PIM II score, were found to be independent predictors of PICU mortality (9–13, 15).

Information is scarce regarding the admission pattern, outcomes, and associated factors of patients admitted to the PICU in developing countries including Ethiopia. PICU settings in Ethiopia are in the infancy stage. while there are attempts to boost the PICU mortality outcome by training PICU staff, preparing PICU admission and discharge criteria furthermore as developing pediatric treatment protocol, we've to attend to work out the effect of such effort. In addition, the previous few studies that were conducted in Ethiopia revealed the presence of wide variation in the reported magnitude of PICU mortality across the different hospitals (9,11). Therefore, the need to conduct additional studies across different settings in the country since such information is very vital to convince stakeholders for planning, training, and prioritizing resource allocation to improve patient outcomes.

Therefore, the current study aimed to assess the admission pattern, treatment outcome, and its associated factor of children who were admitted to PICU of Saint Paul's Hospital Millennium Medical College (SPMMC) over 2 years period.

## Methods

**Setting and study period**: An institutional-based cross-sectional study was conducted at a tertiary hospital, Saint Paul's Hospital Millennium Medical College (SPHMMC), located in Addis Ababa, Ethiopia. It is established in 1969 and it is one of the largest teaching and referral hospitals in Ethiopia with 392 beds, an annual average of 200,000 patients, and a catchment population of over 5 million. The PICU at SPMMC was established in 2016. The Department of Pediatrics and Child Health of the hospital runs PICU with a four-bed capacity which is equipped with four mechanical ventilators. Pediatric patients admitted to the PICU during the period from January 2017 to December 2018 were included in the study. The PICU was staffed with general pediatricians, rotating pediatric residents, one pulmonology critical care fellow, and one par time pediatric emergency and critical care physician. The nurse-to-patient ratio was 1:2. The nurses didn't receive advanced level training in pediatric intensive care (Obtained from SPHMMC pediatrics and child health department).

**Study design and population**: All pediatric patients one month to fourteen years of age and admitted to Saint Paul's Hospital Millennium Medical College PICU during the study period were included. Data were collected through chart review. Charts of pediatric patients who were admitted to the PICU during the study period were retrieved from the record office by using the patient medical registration number which was obtained from the registration logbook found in the PICU.

**Data collection procedures**: Trained data collectors reviewed the following information about the study subjects: age, sex, address, admission diagnosis, admission source, need for mechanical ventilation, duration of a mechanical ventilator, vasopressor use, nutritional status, cardiopulmonary resuscitation, presence of hospital-acquired infection, length of stay, and patient outcome. Charts with incomplete data and patients who stayed less than two hours in the PICU were excluded from the study since there was no adequate time to provide PICU care for such patients.

**Statistical analysis**: The data was analyzed using SPSS version 25.0. Descriptive statistics were used to examine the characteristics of study participants. Logistic regressions with a 95% confidence interval (CI) were utilized to find out the statistical significance. Binary logistic regression was done to identify the crude odds ratio (COR); those variables, which were found significant by COR value was introduced to multiple logistic regression to identify significant associated factors. P < 0.05 was used to declare association. The Hosmer-Lemeshow goodness of fit test was done for the model test and it is fit since the p-value > 0.05.

**For this study, the following operational definitions are used.**

**Length of hospital stay**: is the time from admission to disposition (discharge to the ward or home or death).

**Hospital-acquired infection**: is an infection acquired after 48hr of admission or patient within 7 days of discharge.

**Primary diagnosis**: is the condition established after study to be chiefly responsible for occasioning the admission of the patient to the hospital for care.

**Comorbidity**: chronic or long-term conditions more than one disease or condition is present in the same person at the same time.

**Ethics considerations**: The Institutional Review Board (IRB) of St Paul's Hospital Millennium Medical College provided ethical approval. The ethical clearance was also obtained from Addis Ababa University, College of Health Sciences, departments of Emergency Medicine and Pediatrics, and the child Health research and publication Committee. Additionally, the confidentiality of all the data was seriously respected by not mentioning patients' identifiers in the questionnaire and unauthorized individuals were not allowed to access the data which was collected by using a password-protected computer.

## Results

**Demographic Characteristic of the study participants**: A total of 260 participants were included in this study. In this study, the response rate was 89.7%. About 153 (58.8 %) of the participants were male and the mean age of participants was 48.13 ± 53.65 months, with 153 (58.8 %) of the 260 patients being male (Table 1).

**Table 1:** Sociodemographic characteristic of the patients admitted to SPHMMC PICU (Sept. 2017 to Dec. 2018)

Variable	Category	Frequency (n = 260)	(%)
**Age (year)**	≤1	101	38.7
	1 – 2	31	11.9
	2–5	39	15.0
	5–12	51	19.6
	12–18	33	12.9
**Sex**	Male	153	58.8
	Female	107	41.2
**Region**	Oromia	140	53.8
	Addis Ababa	91	35.0
	Amhara	12	4.6
	SNNPR#	11	4.2
	Tigray	4	1.5
	Others*	2	0.8
**Source of admission**	Emergency Room	136	52.3
	Pediatric Ward	71	27.3
	Home/OPD	50	19.2
	NICU	3	1.2
**Transferred from** **other hospitals**	Public	164	63.1
	Private facility	21	8.1
	Not transferred	75	28.8

* Gambella, Somali, Benishangul, Dire Dawa, Afar

# Southern, Nations, Nationalities, and Peoples' Region

The sources of PICU patient admissions were emergency unit 136(52.3%) patients, ward 71(27.3%) patients, and outpatient department 53(20.4%) patients. The majority of the patients (98.1%) were admitted to the PICU for the first time. Most of the patients were transferred from other public hospitals, 164 (63.1 %), while only 21 (8.1 %) were admitted from private health facilities (Table 1).

Two hundred twenty-six (86.9%) of the patients were admitted for medical reasons and the remaining patients were admitted either for surgical or both surgical and medical conditions. Cough, gastrointestinal complaints, fast breathing, generalized body swelling, and loss of consciousness were the common presentation of the participants in this study, which is 14.6%, 14.6%, 12.7%, 10.8%, and 10.0% respectively. The median duration of presenting symptoms was 4 hours.

The top five primary diseases during admission were Acute Kidney Injury (14.2%), Meningitis (12.3%), Pneumonia (11.5%), Congestive heart failure (10.4%), and Septic shock (7.3%) (Table 2).

**Table 2:** Primary diagnosis upon admission among patients admitted to SPHMMC PICU (Sept. 2017 to Dec. 2018)

Primary diagnosis	Frequency (n = 260)	Percent
Acute Kidney Injury	37	14.2
Meningitis	32	12.3
Severe community-acquired pneumonia	30	11.5
Congestive Heart Failure	27	10.4
Septic Shock	19	7.3
Late-Onset Neonatal Sepsis	14	5.4
Guillain Barre Syndrome	12	4.6
Complicated tuberculosis	11	4.2
Acute on Chronic Kidney Disease	10	3.8
Coma secondary to Poisoning	9	3.5
Acute gastro enteritis with severe dehydration	8	3.1
Diabetic Ketoacidosis	7	2.7
Status Epilepticus	7	2.7
Airway obstruction	5	1.9
Acute Abdomen	4	1.6
Disseminated Staph infection (lung, liver)	3	1.2
Primary Tetanus	3	1.2
Obstructive Sleep Apnea	3	1.2
Severe anemia	3	1.5
Cardiogenic shock	2	0.8
Anaphylactic shock	2	0.8
Perinatal Asphyxia	2	0.8
Acute Asthma Exacerbation	2	0.8
Others *	8	3.2

* Fulminant hepatitis, complicated measles, hypertensive emergency, and TORCH each accounted for 0.8%

Among the infectious primary diagnoses, more than half (56.3%) of commonly affected systems were Neurologic (33.2%) and Respiratory system (23.1%). On the other hand, among the non-infectious cause of primary diagnoses, Cardiovascular, Neurologic, and renal were, 29.2%, 25.0% & 16.7%, respectively.

Overall, only one-third, 83 (31.9%) of the patients had various comorbid illnesses. The frequently identified underlying diseases were congenital heart disease in 10 (3.8%), Down syndrome in 9 (3.5%), chronic renal diseases in 9 (3.5%), and other illnesses. The prevalence of severe acute malnutrition and moderate acute malnutrition were, 13.8% and 21.2%, respectively. Among 223 patients tested for human immunodeficiency virus, 10 (3.8%) were identified positive while 213 were negative.

The overall PICU stay ranged from half a day to 60 days with a mean of 7.26 ±6.87, the median being 6.0 days. The majority of the patients 164 (63.1%) stayed for 2 – 7 days, the other significant proportion of 61 (23.5%) stayed for 8 to 14 days.

Twenty-nine (11.2%) patients were put on a mechanical ventilator and 10 (34.5%) of them died. The mean duration of support lasted 6.4 ± 8.1 days. Among intubated patients, the most frequently used weaning modes were Pressure support (PS) ventilation 17 (6.5%) followed by CPAP only which was employed in 13 (5%). Out of intubated patients, 12 (4.7%) had mechanical ventilation associated complications including 3 (37.5%) ventilator-associated pneumonia, 2 (25.0%) barotrauma, 1 (12.5%) atelectasis along with other complications. Vasopressor agents were used only in 34 (13.1%) of the admitted patients.

**Outcome of patients**: The majority of the patients, 170 (65.4%), were discharged to the pediatric ward while 21 (8.1%) were discharged directly to home and 2 (0.8%) were left against medical advice. In this study, 55 (21.1%) patients died among which 21 (38.2%) were female. The major causes of death were a septic shock in a total of 24 (43.6%), followed by respiratory failure in 14 (25.5%), secondary to a severe chest infection, Guillain Barre Syndrome, and various central causes. Cardiogenic shock accounted for 7 (12.7%).

**Factors associated with patient outcome**: In the univariable analysis, age; sex; PICU length of stay; Mechanical ventilator applied; day of admission; hospital-acquired infection; comorbidity; vasopressor use; CPR during admission; and primary diagnosis during admission were analyzed for the outcome of PICU admitted patients. In multivariable analysis, Cardiopulmonary resuscitations during admission, and need for mechanical ventilation were found to have an association with the outcome of patients (AOR = 16.78 (3.85 – 73.13), CI = 95%: AOR = 2.15 (0.88 – 5.26), CI = 95%, respectively) (Table 3).

**Table 3:** Factors associated with outcome among patients admitted to SPHMMC PICU (Sept. 2017 to Dec. 2018)

Variable	Category	Death		Crude odds ratio (95% CI)	P- value	Adjusted odds ratio (95% CI)	P-value
		Yes	No				
**Age (yr.)**	<1	27	78	3.12(0.68,14.32)	0.15		
	1 to 2	7	24	2.63(0.49, 14.17)	0.26		
	2 to 5	10	36	2.50 (0.50, 12.64)	0.27		
	5 to 12	9	49	1.62 (0.33, 8.40)	0.54		
	12 to 18	2	18	1			
**Sex**	Male	34	119	1.17(0.33,948)	0.61		
	Female	21	86	1			
**Stay (days)**	<1	4	12	1.78 (0.33, 9.48)	0.50		
	2 to 7	38	126	1.61 (0.45, 5.82)	0.47		
	8 to 14	10	51	1.05 (0.26, 4.27)	0.95		
	>14	3	16	1			
**Mechanical** **ventilation**	Yes	10	19	2.03 (1.07, 3.86)	0.03	2.15(0.88,5.26)	0.04
	No	45	186	1			
**Hospital-acquired** **infection**	Yes	7	19	1.76 (0.57, 3.59)	0.45		
	No	48	186	1			
**Day of admission**	Working day	30	109	1			
	Weekend	25	96	1.06 (0.58, 1.92)	0.86		
**Comorbidity**	Yes	18	65	1.05 (0.56, 1.98)	0.89		
	No	37	140	1			
**Vasopressor used**	Yes	10	24	1.68 (0.75, 3.75)	0.21		
	No	45	181	1			
**Cardiopulmonary** **resuscitation on** **admission**	Yes	8	3	11.46(2.93,44.85)	0.001	16.78(3.85,73.13)	0.001
	No	47	202	1			
**Primary diagnosis**	Septic shock	8	11	0.36(0.13, 1.03)	0.06		
	Meningitis	6	26	1.26 (0.46, 3.44)	0.65		
	AKI	5	32	1.86(0.65,5.33)	0.25		
	CHF	3	24	2.33 (0.64, 8.44)	0.20		
	Pneumonia	6	24	0.78 (0.21, 2.54)	0.77		
	LONS	4	14		0.62		
	Others*	23	102	1			

* Guillain Barre Syndrome, Acute gastroenteritis with severe dehydration, Diabetic Ketoacidosis, Airway obstruction, Primary Tetanus, Cardiogenic shock, Status Epilepticus

## Discussion

This study aimed to assess the admission pattern and treatment outcome of patients who were admitted to the PICU of St Paul's Hospital Millennium Medical College, Addis Ababa. Our finding indicated that the mean age of the study population at admission was 48.13 ± 53.65 months and medical conditions (86.9%) were the main reasons for PICU admission. The central nervous system (33.2%) and respiratory systems (23.1%) were the leading organs systems that were affected during admission. Of the total, 11.2% of the patients were mechanically ventilated during their PICU stay and the mortality rate of the PICU was 21.1%.

In the current study, 38.7% of patients were infants which was similar to the study conducted in the PICU of Aga Khan University Hospital (AKUH) Pakistan, which was 37% (16). Similar to the findings by Ayder Hospital, the majority of admitted patients to our PICU were patients with a medical condition (86.9%) (9). In contrast, the study was conducted in the western part of Ethiopia at Jimma University Hospital, where surgical patients make up a large proportion of ICU patients (10). The difference can be explained by the burden of trauma admission to ICU which was high in Jimma referral hospital. The most common reason for ICU admission was infectious (92 %), with meningitis, pneumonia, and sepsis, which was consistent with previous studies in Ethiopia's Gonder and Jimma university hospitals, as well as Tanzania (10,11,17).

The commonly affected organ systems were the Central nervous system (CNS) (33.2%) and respiratory system (23.1%). Correspondingly, the study that was done by Gonder university hospital found out that CNS (31.5%) and respiratory system (10%) were the organ systems commonly affected among patients who were admitted to the PICU (11). The finding of the current study was also similar to the study done in India and Nepal, of which the major cause of admission was reported to be due to CNS and respiratory system involvement (18,19).

The mortality rate in this study was 21.1%. The previously reported mortality rate varied from 2.1 to 41% (10,11,17–20). Because of lack of resources, developing countries have the highest mortality rates (10,18). Even though our patients' mortality was within the reference range, it was relatively high when compared to recent studies in Nepal (19), and Ethiopia (9) which were 12.6%, and 8.5 %, respectively. This is due to a lack of resources, which results in a higher mortality rate, similar to other resource-limited countries. Additionally, our PICU setup is found in one of the tertiary hospitals in the capital city of Ethiopia for which critically ill children are being referred from different parts of the country. In the absence of well-equipped transportation means for critically ill patients, such a poor referral system by itself harms patient outcomes. The lack of trained Pediatric intensivists also could be a reason for the high PICU mortality rate in our setting as reported by a previous systematic review study which showed low-intensity (no intensivist or elective intensivist consultation) ICU was reported to have a higher ICU mortality rate as compared to the high-intensity (mandatory intensivist consultation or closed ICU) groups (21).

Similar to a study done in Gonder (10%), the proportion of mechanically ventilated patients among the total number of admitted patients was (11.2 %). The odds of mortality were 2.15-fold higher in a patient who was in a mechanical ventilator than not being in a mechanical ventilator which is statistically significant (p=0.04). This could be explained by late presentations of patients, the scarcity of both invasive and noninvasive monitoring equipment, and the scarcity of a trained pediatrics intensivist and critical care trained specialty. Similar to studies done in Nepal and Ethiopia (9,20), The odds of developing mortality were 16.78 times higher in a patient who was having post cardiopulmonary resuscitation during admission than who doesn't, which is statistically significant (p = 0.001). This might be because of the absence of organ support monitoring and management for post-cardiac arrest care in the transferring units in the case of our setting.

In conclusion, this study showed the mortality rate was high (21.1%). The most common causes of admission and death were septic shock and infection of the CNS and respiratory system. In this study, post-cardiopulmonary resuscitation admission and use of a mechanical ventilator were statistically significant predictors of mortality.

The higher PICU mortality rate needs action. Studies have shown that well equipping and staffed PICU with intensivists and other trained professionals improve ICU outcomes. Therefore, we recommend the planners and hospital administrators properly look through variables and address the staffing and equipment problems in the PICU of St Paul's Hospital Millennium Medical College.

The limitations of this study include the fact that data were collected through chart abstraction leading to missing values for some of the variables. Also, since the study was a single-center cross-sectional study, the findings may not be generalizable.
